# Acute ischemic STROKE – from laboratory to the Patient’s BED (STROKELABED): A translational approach to reperfusion injury. Study Protocol

**DOI:** 10.1515/tnsci-2022-0344

**Published:** 2024-07-12

**Authors:** Alessandro Sodero, Emilia Conti, Benedetta Piccardi, Cristina Sarti, Vanessa Palumbo, James Kennedy, Anna Maria Gori, Betti Giusti, Enrico Fainardi, Patrizia Nencini, Anna Letizia Allegra Mascaro, Francesco Saverio Pavone, Marzia Baldereschi

**Affiliations:** Neurofarba Department, University of Florence, Viale G. Pieraccini 6, 50139, Florence, Italy; Neuroscience Institute, National Research Council, Via G. Moruzzi 1, 56124, Pisa, Italy; European Laboratory for Non-Linear Spectroscopy, Via Nello Carrara 1, 50019, Sesto Fiorentino, Italy; Stroke Unit, Careggi University Hospital, Largo Brambilla 3, 50134, Florence, Italy; Acute Multidisciplinary Imaging & Interventional Centre, John Radcliffe Hospital, Radcliffe Department of Medicine, University of Oxford, Oxford, United Kingdom; Atherothrombotic Diseases Center, Department of Experimental and Clinical Medicine, University of Florence - Azienda Ospedaliero Universitaria Careggi, Largo Brambilla 3, 50134, Florence, Italy; Neuroradiology Unit, Department of Experimental and Clinical Biomedical Sciences “Mario Serio,”, University of Florence, 50121 Florence, Viale Morgagni 50, 50134, Florence, Italy; Department of Physics and Astronomy, University of Florence, 50019, Sesto Fiorentino, Italy; National Institute of Optics, National Research Council, 50019, Sesto Fiorentino, Italy; Neuroscience Institute, National Research Council, Via Madonna del Piano 10, 50019, Sesto Fiorentino, Italy; Department of Physics and Astronomy, University of Florence, 50019, Sesto Fiorentino, Italy

**Keywords:** stroke, cerebral edema, hemorrhagic transformation, reperfusion injury, biomarkers, translational stroke research

## Abstract

Cerebral edema (CE) and hemorrhagic transformation (HT) are frequent and unpredictable events in patients with acute ischemic stroke (AIS), even when an effective vessel recanalization has been achieved. These complications, related to blood-brain barrier (BBB) disruption, remain difficult to prevent or treat and may offset the beneficial effect of recanalization, and lead to poor outcomes. The aim of this translational study is to evaluate the association of circulating and imaging biomarkers with subsequent CE and HT in stroke patients with the dual purpose of investigating possible predictors as well as molecular dynamics underpinning those events and functional outcomes. Concurrently, the preclinical study will develop a new mouse model of middle cerebral artery (MCA) occlusion and recanalization to explore BBB alterations and their potentially harmful effects on tissue. The clinical section of the study is based on a single-center observational design enrolling consecutive patients with AIS in the anterior circulation territory, treated with recanalization therapies from October 1, 2015 to May 31, 2020. The study will employ an innovative evaluation of routine CT scans: in fact, we will assess and quantify the presence of CE and HT after stroke in CT scans at 24 h, through the quantification of anatomical distortion (AD), a measure of CE and HT. We will investigate the relationship of AD and several blood biomarkers of inflammation and extracellular matrix, with functional outcomes at 3 months. In parallel, we will employ a newly developed mouse model of stroke and recanalization, to investigate the emergence of BBB changes 24 h after the stroke onset. The close interaction between clinical and preclinical research can enhance our understanding of findings from each branch of research, enabling a deeper interpretation of the underlying mechanisms of reperfusion injury following recanalization treatment for AIS.

## 1 Introduction

Despite the progress achieved in the last decade [1,2], stroke remains a leading cause of death and disability worldwide [3,4]. In particular, the short-term prognosis of patients with cardioembolic and atherothrombotic stroke is poor compared with other ischemic stroke subtypes [5] despite the progress achieved in the last decade. Optimal stroke therapy should result in both recanalization of vessel occlusion and reperfusion, i.e., restoration of the downstream capillary blood flow. Most patients who recanalize also reperfuse, but recanalization may result in clinically futile or even detrimental because of the occurrence of reperfusion injury (RI) that is the poor functional outcome, as well as microscopic and macroscopic injury consequential to blood flow restoration [6]. In clinical practice, RI is still unpredictable and includes ischemia expansion, hemorrhagic transformation (HT), and cerebral edema (CE), all of which are associated with the worst clinical outcomes in stroke patients [7]. The literature assessing the post-recanalization period of acute stroke is still scanty but growing, and the pathophysiology and its implications in clinical outcomes are still a matter of debate [8]. Evaluating tissue response after ischemia and reperfusion may expand our understanding of stroke progression. The aim of this study is to evaluate the effects of circulating and imaging biomarkers in relation to CE and HT in stroke patients. In parallel, the preclinical study will develop and characterize a novel mouse model of occlusion and recanalization of the middle cerebral artery (MCA) to investigate BBB alterations and its possible detrimental complications at the tissue level. A close interaction between clinical and preclinical research could lead to a broader understanding of the results deriving from the individual lines of activity, allowing a deeper interpretation of the underlying phenomena. Both the clinical and the preclinical studies constitute an ad hoc investigation on stroke RI. In particular, the aim of our study is:

1) Estimating, in a retrospective longitudinal cohort of patients with acute ischemic stroke (AIS) of the anterior circulation, the role of both imaging and blood biomarkers as predictors of 3-month functional outcome, assessed by modified Rankin Scale (mRS) [9].
2) Using a translational approach, the investigators will develop a new mouse model of light-induced occlusion/reperfusion of the MCA to better reproduce the human setting. Then, the investigators will assess functional impairment induced by stroke with and without recanalization at different time points and the investigators will assess through *ex vivo* experiments the insurgence of blood-brain barrier (BBB) alterations 24 h after the lesion. Finally, the investigators will characterize the stroke volume and the inflammation 1 week after the stroke.
3) Characterizing and quantifying CE and HT in stroke patients by the means of innovative evaluation of routine CT scans. In fact, both CE and HT involve the accumulation of fluids in the brain parenchyma, which in turn produces a distortion of the physiological anatomy: the anatomical distortion (AD).
4) Estimating a large panel of circulating biomarkers as possible predictors of CE and HT occurrence in the clinical cohort.

## 2 Materials and methods

Clinical trial.gov registration: Identifier: NCT05725694.

### 2.1 Clinical section

#### 2.1.1 Clinical setting

All consecutive patients with AIS of the anterior circulation territory who received revascularization therapies (systemic thrombolysis, endovascular treatment) at the Stroke Unit of the Careggi University Hospital from October 1, 2015 to May 31, 2020 are eligible for inclusion (Table 1). After gaining informed consent, individual patient data will be recorded in a dedicated database including demographics and clinical features as well as blood chemistry and neuroradiological parameters. For pathogenesis definition, we will use TOAST classification [10] that denotes five subtypes of ischemic stroke: (1) large-artery atherosclerosis, (2) cardioembolism, (3) small-vessel occlusion, (4) stroke of other determined etiology, and (5) stroke of undetermined etiology. The frequency of various stroke subtypes will be provided in the study population descriptive analyses. Given the retrospective design of the study, it will be possible to evaluate the case-fatality rate, including in-hospital mortality. Blood biomarkers and neuroimaging features will be collected on arrival at the hospital and repeated 24 h following intervention will be included in the analysis. The functional outcome will be assessed 3 months after stroke according to the mRS [9] by outpatient visit or phone interview.

**Table 1:** Inclusion and exclusion criteria in the STROKELABED study Clinical section

Inclusion criteria	Exclusion criteria
Patients providing informed consent to data processing	Patients denying consent to data processing
Stroke in the anterior circulation territory	Stroke in the posterior circulation
Patients consecutively admitted to Careggi University	Pregnancy
Hospital in Florence	
Patients with available head CT scan in DICOM format performed before the acute phase treatment and at 24 h follow up	Age <18 years

#### 2.1.2 Laboratory protocol

The protocol previously documented [11] will be followed, where blood samples were routinely collected in AIS patients, before recanalization treatment and after 24 h. Whole venous blood is collected in tubes without anticoagulant. Tubes are centrifuged at room temperature at 1,500 g for 15 min, and the supernatants are stored in aliquots at −80°C until measurement of inflammatory markers, Matrix Metalloproteinases (MMPs) and Tissue inhibitors metalloproteinases (TIMPs). Levels of different inflammatory markers (IL-4, IL-6, IL-10, IL-12, alpha2-macroglobulin, Myeloperoxidase, CXCL-8, CXCL10 VEGF, ICAM-1, VCAM-1), zinc-dependent endopeptidases and their inhibitors (MMP-2, -3, -7, -8, -9, TIMP-1, -2, -3, -4 and EMMPRIN) will be determined using Bio-Plex suspension array system and R&D Kits (R&D System, Milan, Italy) following manufacturer’s instructions.

#### 2.1.3 Neuroimaging protocol

A complete neuroimaging assessment, including non-contrast CT (NCCT), CT-angiography (CTA), and CT-perfusion (CTP) is performed at hospital arrival before acute interventions, according to guidelines in place at the time of treatment [12,13]. All patients undergo a 24-h follow-up NCCT. All available neuroimaging will be exported in DICOM format in order to be processed and analyzed and then will be stored in the electronic database.

The presence of HT will be defined according to the European Cooperative Acute Stroke Study (ECASS) II on 24 h NCCT brain scan. HI1: Hemorrhagic infarction type 1; HI2: Hemorrhagic infarction type 2; PH1: Parenchymal hematoma type 1; and PH2: Parenchymal hematoma type 2 [14]. All uncertain cases will be resolved by an experienced neuroradiologist.

Two trained neurologists will independently evaluate each patient’s 24-h NCCT and assess the presence of CE classified according to CE classification used by the Helsinki Stroke Thrombolysis Registry Group [15], where

- CED1 = Focal brain swelling up to one-third of the hemisphere;
- CED2 = Focal brain swelling greater than one-third of the hemisphere;
- CED3 = Brain swelling with midline shift;
- NONE = absence of cerebral edema.

The occurrence of CE and HT after AIS will be also investigated by the means of innovative neuroimaging evaluations to obtain a quantitative and reliable measure, based on the AD concept: both CE and HT imply accumulation of fluid within cerebral tissue creating a brain swelling, which distort normal anatomy of brain itself. Corrected infarct and AD volume will be calculated [16] according to the following outline methodological steps all using FMRIB Software Library v6.0 software (created by the Analysis Group, FMRIB, Oxford, UK):

a) The ischemic lesion on the 24 h NCCT will be masked independently by two neurologists. The Dice similarity index will be calculated for the two masks. For those with a Dice similarity index greater than 0.8, an intersection mask will be generated for subsequent analysis. For those with a Dice similarity index less than 0.8, the mask will be reviewed and finalized by a staff neuroradiologist.
b) The follow-up NCCT scan will be registered to the baseline NCCT scan using FMRIB’s Linear Image Registration Tool, and, FMRIB’s Nonlinear Image Registration Tool [17]. The lesion masks will be resampled on the registered follow-up scans using either the nonlinear warp or the linear matrix generated with a threshold of 0.5 applied. All masks and registrations will be individually validated to ensure optimal registration.
c) Final infarct volume will be defined as the volume of the infarct mask after nonlinear registration. AD will be calculated as the difference in the volume of the infarct masks generated after linear and nonlinear registration to the baseline NCCT.

### 2.2 Clinical outcome measures

#### 2.2.1 Months functional outcome

The mRS will be adjudicated in all enrolled patients 3 months following stroke onset [9] either in person from clinic visit or by telephone interview where the patient is unable to attend a clinic in person. The occurrence of CE. Time frame: NCCT brain scans acquired at 24 h after the stroke event (see Neuroimaging protocol). The occurrence of HT. Time frame: NCCT brain scans acquired at 24 h after the stroke event (see Neuroimaging protocol).

### 2.3 Preclinical section

In the preclinical research, we will characterize the behavioral impairment and the occurrence of alterations of BBB permeability in the acute phase after stroke in a novel mouse model of occlusion and recanalization of the distal branch of the MCA. Moreover, we will quantify the infarct growth and progression of the inflammatory response both in the ipsilateral and contralateral hemispheres. Blinded experiments and *in vivo* and *ex vivo* analyses will be conducted.

#### 2.3.1 Mouse model of MCA occlusion and recanalization

In this project, we will apply a newly developed mouse model of stroke targeted to the para-orbital branch of the MCA [18]. Mice will be anesthetized with ketamine (50 mg/kg) and xylazine (9 mg/kg), and body temperature will be monitored and maintained at 37°C with a heating pad (ThermoStar Temperature Controller, RWD, USA). To induce the occlusion of the blood vessel, we will perform a small craniotomy on the squamosal bone and then, after an intraperitoneal injection of the photosensitive dye Rose Bengal, we will illuminate the MCA with a green laser. The dye activation will induce the formation of oxygen free radicals, causing endothelial damage that triggers platelet aggregation and clot formation. After defined periods of stable occlusion (30, 60, and 90 min), we will illuminate the occlusion site with a UV LED capable of disrupting the fibrine bounds inside the clot resulting in the recanalization of the blood vessel.

#### 2.3.2 Behavioral evaluation

Functional impairment induced by the occlusion of the distal MCA with and without recanalization will be assessed according to a panel of different motor tests to assess strength, balance, adduction, and flexion capability modifying the protocol applied by Balbi and collaborators [19], based on a standardized qualitative assessment for measuring the degree of motor impairment [20]. The experimental protocol will include three-time points: pre-stroke, 24 h, and 5 days after the damage. This will allow the longitudinal comparison of motor performance in the pre-stroke, acute, and subacute phases.

#### 2.3.3 Characterization of BBB leakage

We will assess the presence of alterations in BBB permeability 24 h after the insult through *ex vivo* experiments. To this aim, we will inject the Evans Blue dye in the mouse tail vein, characterized by a very high affinity for serum albumin. In physiological conditions, serum albumin cannot pass through the BBB. Where BBB integrity is lost, the Evans Blue dye will cross into the brain parenchyma and stain the brain tissue.

#### 2.3.4 Investigation of tissue-damaged extension

We will evaluate the presence of necrotic tissue 7 days after ischemia through *ex vivo* experiments, as previously described by Conti et al., [21]. After anesthetizing the animals with an overdose of ketamine (50 mg/kg) and xylazine (9 mg/kg), mice will be transcardially perfused with 4% paraformaldehyde to allow tissue fixation. The brain will then be cut into 100 µm thick coronal sections with a vibrating-blade vibratome (Leica, Germany) to perform immunostaining of the Neuronal marker, NeuN (1:1,000, anti-NeuN chicken, Millipore, Germany). The stroke volume for each animal will be calculated by summing up all damaged areas, characterized by the absence of immunostaining, and multiplying the number by section thickness and by 4 (the spacing factor). Images will be acquired with a Stemi 508 (Carl Zeiss). The total volume in mm^3^ will be given as the mean value ± standard error of all analyzed animals.

#### 2.3.5 Reactive astrogliosis characterization

The emergence of the acute inflammatory response associated with the upregulation of the glial fibrillary acidic protein (GFAP) in astrocytes will be assessed. The number of GFAP-positive neurons will be analyzed using a confocal fluorescence microscope (Nikon Eclipse TE 300, Tokyo, Japan) with a Nikon Plan EPO 60X objective (NA 1.4, oil immersion Nikon, Tokyo, Japan). We will focus our investigation on four regions of interest, i.e., the peri-infarct area (ischemic border zone), a region in the ipsilesional hemisphere distant to the stroke core (remote zone in the ipsilesional hemisphere), a region in the contralateral hemisphere homologous to the ischemic core, and a region in the contralateral hemisphere homologous to the periinfarct area.

### 2.4 Statistical analysis

#### 2.4.1 Clinical section

We will divide the study cohort into two groups according to the outcome to be explored: functional outcome (mRS0-2 vs mRS 3-6), CE occurrence (CE yes vs CE no), relevant HT occurrence (HI-2, PH-1 or PH-2 vs HI-1 or no HT). Data will be presented as mean value (standard deviation, SD), median (interquartile range, IQR), and number (percentage) as appropriate. The levels of all circulating biomarkers will be recorded as continuous variables. We will describe the general characteristics of the population and use Student’s *t*-test, the Mann–Whitney *U*-test, and Pearson’s chi-squared test, as appropriate, to test the differences between groups. Moreover, logistic regression analysis will be used to calculate odds ratio (OR) for each outcome under investigation with 95% confidence intervals (CI) adjusting for age, gender, stroke severity (according to the National Institutes of Health Stroke Scale, NIHSS), AD, BBB leakage (according to CTP K trans value > 0.63), history of hypertension, diabetes, atrial fibrillation, taking anticoagulants or antiplatelets, type of treatment (Thrombolysis rt-PA, endovascular, and combined). The presence or absence of systemic inflammation will be assessed by a proxy measure: current adherence to corticosteroid therapy. Analyses will be restricted to patients with complete data. The study design is retrospective and the data collection process is embedded in the usual clinical routine for AIS patients at the single center of Careggi University Hospital of Florence, Italy; therefore, it is likely that missing data could be few and completely at random, resulting in unbiased estimates. For all statistical analyses, a two-sided *p*-value < 0.05 will be considered as statistically significant. Analyses are performed using IBM SPSS Statistics for Windows, Version 23.0 (IBM CO. Armonk, NY).

#### 2.4.2 Preclinical section

All the analyses of both *in vivo* and *ex vivo* experiments will be blinded. Moreover, all the data will be independently evaluated by the two researchers who perform the experiments and the analysis. Results will be considered statistically significant if their corresponding P value will be less or equal to 0.05. OriginPro software (OriginLab Corporation) will be used for all other statistical analyses. For all ANOVAs that prove statistically significant, multiple comparisons among time points and different regions of the cortex will be assessed using the ANOVA Repeated Measures followed by a post hoc Tukey HSD test.

Mice will be housed in clear plastic cages under a 12 h light/dark cycle and given ad libitum access to water and food. We will use a transgenic mouse line, C57BL/6J-Tg(Thy1-EGFP)MJrs/J, from Jackson Laboratories (Bar Harbor, Maine USA).


**Ethics statement clinical research:** The research related to human use has been complied with all the relevant national regulations, institutional policies and in accordance with the tenets of the Helsinki Declaration, and has been approved by the authors’ institutional review board or equivalent committee. The study has been approved by the local Ethics Committee (ethics committee registration number: Comitato Etico Area Vasta Centro [CEAVC] 16923_oss).
**Informed consent statement: **Informed consent has been obtained from all individuals included in this study.
**Ethical approval:** The research related to animals’ will be complied with all the relevant national regulations and institutional policies for the care and use of animals. All procedures involving mice will be performed in accordance with regulations of the Italian Ministry of Health authorization n. 723/2019.

## 3 Discussion

To the best of our knowledge, this is the first study specifically targeting stroke RI both from a clinical and a preclinical perspective. CE and HT are usually evaluated using qualitative rating scales that inadequately reflect the variability and complexity of the underlying biological phenomena, [22,23,24]. Some quantitative approaches are limited by the arbitrary measurement of the two phenomena separately (CE and HT), which does not reflect the fact that both occur on an indistinguishable biological continuum [25]. This flawed approach to measuring RI manifestations has constrained the ability to detect reliable predictors across studies [23,26,27].

In this project, the occurrence of CE and HT after AIS will be also investigated on routine CT scans by the means of modern evaluation techniques to obtain quantitative and reliable measures of these potentially adverse phenomena. We aim to evaluate the correlation of those radiological features with clinical features and circulating blood biomarkers related to BBB disruption in order to identify predictors of RI in patients undergoing recanalization therapies.

Moreover, we hypothesize that possible interactions between circulating biomarkers may provide clues to prevent and counteract reperfusion injury. To investigate possible tissue mechanisms underpinning RI we will focus on inflammation and extracellular matrix biomarkers that are reported to degrade the tight junctions of the BBB, resulting in cell leakage and extracellular matrix degeneration, leading to CE and HT [28]. Edema formation and HT are often erroneously considered as two separate phenomena and typically not included in the same spectrum of events. These vascular complications after stroke derive basically from the same pathological response to ischemia and should be hence taken into account together. In addition, beyond molecular considerations and clinical implications, CE and HT are strongly linked also in the neuroimaging field. Both phenomena imply the accumulation of fluid within cerebral tissue, therefore creating brain swelling, which distorts the normal anatomy of the brain itself. The “anatomical distortion” concept includes both CE and HT and is related to their damaging consequences on tissue and on patients’ outcomes. BBB disruption is a major pathological aspect of ischemic stroke and is regulated by different factors working at different stages of cerebral ischemia. These components interact worsening ischemic brain injury and increasing the propensity to the occurrence of CE and HT. Ischemia triggers the neuroinflammation cascade, while the oxidative stress induces MMPs released by neurons, glia, astrocytes, and pericytes causing BBB damage through degradation of the endothelial basal lamina [29]. We therefore have chosen to investigate several neuroinflammation biomarkers and MMPs together with the TIMPs.

In parallel with the clinical study, we will exploit a novel mouse model of stroke to investigate the underlying aspects of these processes, understanding the relationship between neuronal degeneration, alterations of vascular permeability, and the inflammatory response at the organ scale. This development of a mouse model of stroke capable of reproducing human large vessel occlusion represents a great potential value for translational research with the generation of preclinical datasets suitable for investigating both the fine mechanisms underlying structural BBB modifications [30] and post-stroke alterations of functional connectivity [31,32,33,34,35].

Some limitations of the study must be acknowledged. For the clinical section, the lack of a control group of AIS patients not treated with revascularization might underestimate biomarkers’ predictive role. The correct identification of the ischemic lesion in posterior circulation stroke on head NCCT can often be uncertain and challenging. Given the need to manually delineate the lesion as the basis for measuring AD, and given the greater tendency to present method artifacts (intrinsically linked to the small size of the brain posterior regions and the close presence of the surrounding bone), we decided to limit inclusion to patients with anterior circulation stroke, to obtain more accurate and reliable image processing. In addition, since a worse prognosis often characterizes posterior circulation strokes, the exclusion of such patients allows us to obtain a more homogeneous cohort to estimate the role of predictive factors of RI more accurately. Furthermore, it is improbable that we could explore differences according to the territory of artery occlusion [36]. In fact, this is an observational study that will enroll consecutive patients over a specific period; it will therefore be unlikely to reach a sufficient number of anterior cerebral artery stroke patients to guarantee reliable estimates in the comparison with MCA stroke patients. Moreover, stroke heterogeneity in humans (stroke severity, location, comorbidity, age, systemic inflammation before the stroke, etc.) might determine variabilities of biomarkers’ levels producing inconsistent findings. We will try to overcome this problem by evaluating the differences between baseline and 24-h levels of each biomarker.

Regarding preclinical research, despite the significant efforts of preclinical researchers to develop animal models of cerebral ischemia that accurately reflect human pathology, it remains highly challenging to account for the wide range of biological variables (such as age, sex, comorbidities) present in stroke patients (see review by Conti et al., [37]). Nevertheless, we believe that a well-designed animal model can reveal crucial aspects of the underlying pathophysiological mechanisms.

The STROKELABED study stems from a multidisciplinary research group [37] (Translational REsEarch on Stroke, TREES) that is carrying out a joint research on patients and animal models, taking advantage of a valuable cross-fertilization between clinical and preclinical research, aiming at providing novel insights into unmet clinical stroke needs.

## 4 Conclusion

Stroke has heterogeneous pathophysiologies even among patients with the same stroke mechanisms, and therefore a personalized approach is needed, based on precision medicine, which in turn is based on biomarker profiling. This stimulated a joint clinical–preclinical approach to exploit stroke research to move beyond recanalization to predict RI. Both preclinical and clinical research are still looking for drugs that can antagonize RI with a good efficacy and safety profile, similar to what has recently happened for the glenzocimab for the no-reflow phenomenon [38,39]. Mechanisms of inflammation are rather complex, therefore it is unlikely that a single control of a single molecule can achieve the control of the entire process underpinning stroke RI. What is needed is a nuanced approach to target BBB dysregulation during the acute phase of injury, without interfering with later brain tissue repair. We hope that our translational study can pave the way for more robust and impactful research in the future. The major strength of our study lies in the parallel clinical/preclinical approach. In future studies, to enhance the translational significance of our research, we will evaluate a panel of circulating biomarkers in mice that are also analyzed in stroke patients. Additionally, we will characterize the occurrence of CE by assessing brain water content in injured brains. Finally, we will use high-resolution two-photon imaging to investigate the structural plasticity of axons and dendrites in the acute phase after stroke.

The close interaction between clinical and preclinical research could lead to a broader understanding of the results deriving from the individual branches of research activity, allowing a deeper interpretation of the underlying phenomena of RI post-recanalization treatment for AIS.
